# Foliar Application of Chitosan Nanoparticles Mitigates Early Physiological and Antioxidant Responses of *Solanum lycopersicum* L. Seedlings Under Mild-to-Moderate Water Deficit

**DOI:** 10.3390/polym18111275

**Published:** 2026-05-22

**Authors:** Ricardo Tighe-Neira, Gonzalo Tortella-Fuentes, Verónica Véjar-Cayuqueo, Emilio Jorquera-Fontena, Jorge González-Villagra, Rafael J. V. Oliveira, Felipe L. N. Sousa, Bianca G. P. Araújo, Rodrigo Rodríguez, Claudio Inostroza-Blancheteau

**Affiliations:** 1 Laboratorio de Fisiología y Biotecnología Vegetal, Departamento de Ciencias Agropecuarias y Acuícolas, Facultad de Recursos Naturales, Universidad Católica de Temuco, Temuco 4780000, Chile; 2 Núcleo de Investigación en Producción Alimentaria, Facultad de Recursos Naturales, Universidad Católica de Temuco, Temuco 4780000, Chile; 3 Centro de Excelencia en Investigación Biotecnológica Aplicada al Medio Ambiente (CIBAMA-BIOREN), Universidad de La Frontera, Casilla 54-D, Temuco 4780000, Chile; 4 Escuela de Agronomía, Facultad de Ciencias, Ingeniería y Tecnología, Universidad Mayor, Temuco 4801043, Chile; 5 Centro para la Resiliencia, Adaptación y Mitigación (CReAM), Universidad Mayor, Av. Alemania 281, Temuco 4801043, Chile; 6 Centro de Tecnologías Estratégicas de Nordeste (CETENE), Recife 50740-540, PE, Brazil; rafael.oliveira@cetene.gov.br (R.J.V.O.);; 7 Instituto de Ciencias Aplicadas, Facultad de Ingeniería, Universidad Autónoma de Chile, Temuco 4780000, Chile; 8 ANID–Millennium Science Initiative Program–Millennium Nucleus in Data Science for Plant Resilience (Phytolearning), Santiago 8370186, Chile

**Keywords:** chitosan, foliar application, *Solanum lycopersicum*, oxidative stress, drought stress

## Abstract

*Solanum lycopersicum* is highly sensitive to water deficits, which negatively affect photosynthesis and increase oxidative stress. Although chitosan nanoparticles (ChNPs) offer a sustainable solution, research on their effects on this species is scarce. This study evaluated whether ChNPs mitigate the physiological and biochemical effects of water deficit on *S. lycopersicum* seedlings. Thirty-day-old seedlings were grown under greenhouse conditions, and two irrigation levels were established: 80% of substrate water-holding capacity (well-watered, WW), and 50% of water-holding capacity (mild-to-moderate water deficit, WD). Spherical ChNPs with a size of 39.52 ± 10.9 nm were suspended in 1% acetic acid and foliar-applied at 0, 60, or 120 mg L^−1^. After 10 days, biomass accumulation, chlorophyll fluorescence parameters (F_v_′/F_m_′, ΦPSII, and ETR), gas exchange, and non-enzymatic antioxidant traits were determined. Even under this early-stage stress regime, water deficit significantly reduced shoot and root biomass, net photosynthesis, and stomatal conductance, while increasing lipid peroxidation. Foliar application of ChNPs, particularly at 60 mg L^−1^, restored dry matter production and improved photochemical efficiency and electron transport rate by 14%; likewise, net CO_2_ assimilation increased by 11.7%. In addition, this dose enhanced antioxidant activity and total phenols by 66% and 1.6-fold, respectively. ChNPs at 60 mg L^−1^ mitigated the effects of WD in *S. lycopersicum* by increasing antioxidant and photosynthetic performances. Nevertheless, additional molecular studies, including enzymatic antioxidant characterization and compatible solute profiling, are required to elucidate the mechanisms involved.

## 1. Introduction

Water deficit is the major abiotic constraint for global crop production [1], and Satoh et al. [2] anticipate that such events will increase across multiple regions worldwide by the end of the 21st century. In this context, while Latin America holds a substantial portion of the world’s freshwater reserves, hydrological disparities lead to severe water scarcity across several nations. Specifically, Chile, México, and Perú are increasingly experiencing prolonged and recurrent droughts [3,4], which impose significant constraints on agricultural productivity and, consequently, on the socio-economic and cultural fabric of these regions. Therefore, identifying effective strategies to counteract water-induced stress in agriculture is not only necessary but a pressing priority for ensuring food security and regional stability [5]. Tomato (*Solanum lycopersicum* L.) is an important crop globally, with pivotal roles in food and commercial use [6,7]. This species is considered a natural source of antioxidants due to its high concentration of lycopene, carotenoids, and anthocyanins [7,8]. According to the Food and Agriculture Organization of the United Nations [9], global production of *S. lycopersicum* exceeded 250 million tons in 2024, with more than 6.2 million hectares harvested. From an economic perspective, according to data from the Observatory of Economic Complexity [10], in 2024, the trade balance for tomatoes totaled $11.9 billion, with México, the Netherlands, and Morocco as the largest exporters. However, *S. lycopersicum* is highly susceptible to water deficit, a stress that causes decreases in physiological, biochemical, morphological, yield, and quality parameters [6,11,12]. Indeed, water deficit directly affects the reproductive phase, causing flower abortion and limited fruit set, thereby leading to substantial reductions in tomato yield and quality [13,14,15]. It has been reported that commercial genotypes of *S. lycopersicum* suffer yield reductions of up to 26% under water-deficit conditions (50% field capacity) [16].

Water deficit significantly impairs photosynthesis and growth in several species [7,17,18,19]. In the photochemical phase of photosynthesis, lower values were reported for the maximum quantum yield (F_v_/F_m_), the effective quantum yield (Φ_PSII_), the electron transport rate (ETR), and photochemical quenching (qP), and an increase in the non-photochemical quenching (NPQ) was noted in *S. lycopersicum* compared to the control [7,20,21,22]. In terms of gas exchange, *S. lycopersicum* initially responds to water deficit by closing its stomata, which reduces stomatal conductance (*g*_s_) and net photosynthesis (P_n_). However, during severe water deficit, non-stomatal mechanisms are also activated, further decreasing P_n_ [21].

Secondary metabolism is a natural response in plants to biotic or abiotic stress, helping counteract the generation of reactive oxygen species (ROS) and the resulting oxidative stress. Some studies have reported that *S. lycopersicum* enhanced both enzymatic and non-enzymatic antioxidant defense systems under water deficit [7,23]. This includes increased total antioxidant capacity, polyphenol, flavonoid, and proline concentrations. Consequently, as water deficit intensifies, malondialdehyde content increases, reflecting oxidative stress [21].

Chitosan NPs (ChNPs) are a cationic, linear copolymer consisting of randomly distributed β (1→4)-linked units of 2-amino-2-deoxy-D-glucose (D-glucosamine) and 2-acetamido-2-deoxy-D-glucose (N-acetyl-D-glucosamine) [24]. Chitosan, a natural biopolymer, is a natural biostimulant that can promote natural defense mechanisms against various agents, potentially paving the way for sustainable and precision agriculture [25,26]. However, their solubility under acidic conditions, molecular weight variability, and degree of deacetylation are important parameters that must be validated when transitioning from the greenhouse to the field. In *S. lycopersicum*, ChNPs have mainly been studied under heavy metal stress, where their application increased biomass productivity (shoot dry weight increased by 38%) and gas exchange (P_n_, *g*_s,_ and transpiration (*E*)) [27]. Furthermore, the authors observed an increase in the following photochemical parameters of photosynthesis: F_v_/F_m_, Φ_PSII_, and NPQ. They also observed increased antioxidant activity of polyphenol oxidase, peroxidase, catalase, and ascorbate peroxidase, and a consequent reduction in lipid peroxidation. The effects of ChNPs on water deficit have been previously reported in non-crop species, such as *Lupinus termis* and *Catharanthus roseus* (L.), in which enhanced tolerance was observed in both cases. This improvement in water-deficit tolerance induced by ChNPs was achieved through an integrated plant response, including increases in growth, yield, photosynthetic pigments, proline, free amino acids, and *g*_s_ [28,29]. ChNPs are thus a promising solution for combating the effects of water deficit because, unlike many other alternatives, they do not introduce synthetic elements. This makes them a more sustainable option and enables their use at various stages of crop growth [30,31]. However, the potential role of ChNPs in alleviating water deficit in *S. lycopersicum* remains largely unexplored.

Of particular agronomic relevance is the early seedling stage, during which mild-to-moderate water restriction can establish physiological trajectories that determine later crop performance. Understanding plant responses at this stage, and how biostimulants such as ChNPs can intercept them, is thus essential for developing preventive rather than reactive crop management strategies.

Despite the growing interest in ChNPs as biostimulants, their potential to mitigate water deficit in *S. lycopersicum* remains largely unexplored. Existing studies have primarily focused on heavy metal stress in this species, whereas research on ChNPs-water deficit interactions has been limited to non-crop species. Furthermore, no study to date has evaluated the early physiological and antioxidant responses of *S. lycopersicum* seedlings to foliar ChNPs application under mild-to-moderate water deficit, a condition of direct agronomic relevance for early crop management. Addressing this gap is essential for developing preventive, sustainable strategies to support *S. lycopersicum* production in water-scarce regions. We hypothesized that ChNPs enhance drought resilience by optimizing photosynthetic efficiency and upregulating the antioxidant defense system, thereby mitigating oxidative damage and preserving biomass accumulation under water deficit. Thus, this study evaluated whether ChNPs mitigate the physiological and biochemical effects of water deficit on *S. lycopersicum* seedlings, providing insights into the use of ChNPs as a sustainable strategy for engineering crop resilience in water-scarce regions.

## 2. Materials and Methods

### 2.1. Characterization of Chitosan Nanoparticles

The chitosan nanoparticle samples of 63.5 kDa were purchased from Nanotec S.A., and their structure and interfaces were characterized. For this purpose, the structure of chitosan powders was investigated using X-ray diffraction (XRD) patterns obtained with an X-ray diffractometer, Rigaku SmartLab SE (Rigaku Corporation, Tokyo, Japan), equipped with Cu Kα radiation (λ = 1.540598 Å) and a HyPix-400 detector, in the 2θ range from 5° to 40° with steps of 0.01°. To determine the size of chitosan crystallites, the Scherrer equation was applied, expressed by D = Kλ/βcosθ, where D is the size of crystallites (nm), K is the dimensionless Scherrer shape factor with value of 0.9, λ is the wavelength of the incident X-ray beam of the diffractometer (Ka Cu 1.540598 Å).

The morphology of chitosan powders was analyzed using Scanning Electron Microscopy (SEM) images obtained with a Mira 4 SEM Microscope (TESCAN, Brno, Czech Republic) equipped with an FEG Schottky electron emission source and operated at an acceleration voltage of 5 kV. The composition of the samples was investigated using an Energy-Dispersive X-ray spectroscopy (EDX) detector (Oxford Instruments®, Oxford, UK) coupled to an SEM. The chitosan powder samples were deposited onto the aluminum/nickel tape, which was fixed in the holder. The chitosan nanostructure was investigated using an FEI Morgagni 268D transmission electron microscope (TEM) (FEI, Brno, Czech Republic) operating at an acceleration voltage of 100 kV. The nanostructures were suspended in an aqueous acetic acid solution (1%, *v*/*v*) and homogenized using a low-power ultrasound device. The solution was diluted 1:10 (*v*/*v*) in deionized water and applied to a 400-mesh holey carbon-coated Cu grid. The median size and size distribution data were obtained from TEM images, with measurements taken from at least 500 randomly selected nanoparticles using ImageJ software (version 1.52b, National Institutes of Health, Bethesda, MD, USA). Fourier transform infrared (FTIR) spectroscopy was employed to investigate the functional groups present in chitosan. A spectrum was acquired on a Bruker Vertex 70 spectrophotometer using attenuated total reflection (ATR), with a resolution of 4 cm^−1^ over 16 scans in the 4000–400 cm^−1^ range.

### 2.2. Plant Material and Growth Conditions

Thirty-day-old Cal Ace tomato (*Solanum lycopersicum* L.) seedlings were transplanted into one-liter containers filled with a mixture of soil (Andisol), peat, and perlite at a ratio of 2:2:1 (*v*:*v*:*v*), which contained N-P_2_O_5_-K_2_O 15-12-29 and 1.0 kg m^−3^ of microelements. Each replicate consisted of one container with a single plant. After transplanting, seedlings were conditioned in a greenhouse for 7 days. The experiment was carried out in a polycarbonate greenhouse under the following conditions: temperature 23 ± 2 °C, relative humidity 60–80%, photosynthetic photon flux density (PPFD) 600 μmol photons m^−2^ s^−1^, and a photoperiod of 16/8 h light/dark.

### 2.3. Chitosan Nanoparticle Suspension

The chitosan nanoparticle suspension was prepared from commercially available samples using the method proposed by Behboudi et al. [32] to determine the doses. Briefly, 1 g of ChNPs was suspended in 100 mL of 1% acetic acid under constant stirring. Doses of 60 and 120 mg L^−1^ were then prepared from this concentrated suspension, with the pH adjusted to 6 using NaOH.

### 2.4. Treatments and Measurements

After the conditioning period, *S. lycopersicum* seedlings were subjected to two irrigation levels: (1) plants irrigated at 80% of substrate water-holding capacity (well-watered, WW) and (2) plants irrigated at 50% of water-holding capacity (WHC), considered as water deficit (WD), based on the study of Machado et al. [33]. In addition, ChNPs were foliar applied at 0, 60, or 120 mg L^−1^. Therefore, treatments were as follows: (1) well-watered plants without ChNPs (WW-Control), (2) well-watered plants with 60 mg L^−1^ ChNPs (WW-60), (3) well-watered plants with 120 mg L^−1^ ChNPs (WW-120), (4) plants subjected to water deficit without ChNPs (WD-Control), (5) plants subjected to water deficit with 60 mgL^−1^ ChNPs (WD-60), and (6) plants subjected to water deficit with 120 mg L^−1^ ChNPs (WD-120). Ten days after the application of ChNPs, photosynthesis and chlorophyll fluorescence were measured in vivo, and leaf tissue samples were harvested. This tissue was frozen in situ using liquid nitrogen and stored at −80 °C in an ultra-freezer until biochemical analyses were performed.

### 2.5. Chlorophyll Fluorescence and Gas Exchange

The photochemical efficiency of PSII (F_v_′/F_m_′), the effective quantum yield of PSII (Φ_PSII_), and the electron transport rate (ETR) were determined under light-adaptation conditions using the formulas proposed by Maxwell and Johnson [34]. F_v_′/F_m_′ = (F_m_′ − F_o_′)/F_m_′, Φ_PSII_ = (F_m_′ − F_s_′)/F_m_, and ETR = PPF × 0.5 × Φ_PSII_ × 0.84. Measurements were taken between 09:00 and 11:00 h using a fluorescence chamber (6400-40) coupled with an infrared gas analyzer (IRGA, LI-6400XT, LI-COR, Lincoln, NE, USA).

The same equipment was used to measure gas exchange, expressed as net photosynthesis (P_n_), stomatal conductance (*g*_s_), and transpiration (*E*). Additionally, intrinsic water use efficiency (WUE) was calculated as P_n_/*g*_s_ [35]. The measurements were performed between 09:00 and 13:00 h under the following conditions: irradiance of 600 μmol photons m^−2^ s^−1^, temperature of 20 °C, relative humidity of 80%, CO_2_ concentration of 400 μmol mol^−1^, and a flow rate of 200 cm^3^ min^−1^.

### 2.6. Photosynthetic Pigments

The amount of photosynthetic pigments were determined according to the protocol of Lichtenthaler and Wellburn [36]. For this, plant tissue (0.1 g) was pulverized in liquid nitrogen. Then, CaCO_3_ and 1 mL of 100% methanol were added. The resulting mixture was transferred to an amber Eppendorf tube and centrifuged at 10.000× *g* for 5 min at 4 °C. The supernatant was transferred to a new amber Eppendorf tube. The same process was repeated with the obtained pellet, after which the two resulting supernatants were combined. A total of 750 µL of 100% methanol and 250 µL of the extracted supernatant were added for the measurement. The absorbances were measured at 666 nm for chlorophyll *a* and 653 nm for chlorophyll *b* using a Thermo Scientific Spectronic Genesis 10 UV-Vis scanning spectrophotometer (Madison, WI, USA). In addition, the total chlorophyll content (Chl *a* + Chl *b*) and the chlorophyll a/b ratio were determined.

### 2.7. Antioxidant-Related Parameters

For analysis of antioxidant activity and total phenol levels, 0.1 g of leaf tissue was pulverized in liquid nitrogen and macerated with 1 mL of 80% methanol (*v*/*v*). Then, the homogenate was transferred to 1.5 mL Eppendorf tubes and centrifuged at 13.000× *g* for 5 min at 4 °C. The resulting supernatant was transferred to amber tubes and stored until analysis.

Antioxidant activity was determined using the 2,2-diphenyl-1-picrylhydrazyl (DPPH) radical method, as described by Chinnici et al. [37]. For this purpose, 4 µL of the supernatant, 196 µL of 80% methanol, and 800 µL of DPPH were mixed. After incubating the samples for 8 min at room temperature, absorbance was measured at 515 nm with a Thermo Scientific Spectronic Genesis 10 UV-Vis Scanning Spectrophotometer (Madison, WI, USA), using Trolox as the standard. The results were expressed as Trolox equivalent per gram of fresh weight (TE [mg g^−1^ FW]), according to the regression curve formula y = 0.0754x + 0.0133 (R^2^ = 0.9905).

Total phenols were determined using the Folin–Ciocalteu method [38]. The absorbance was determined at 765 nm using a Thermo Scientific Spectronic Genesis 10 UV-Vis Scanning Spectrophotometer (Madison, WI, USA), with chlorogenic acid used as the standard. Total phenolic content was expressed as milligrams of chlorogenic acid equivalents per gram of fresh weight (mg CAE g^−1^ FW).

### 2.8. Lipid Peroxidation

Lipid peroxidation was determined by measuring thiobarbituric acid-reactive substances (TBARS) using a modified protocol [39]. Leaf samples were macerated with a mixture of trichloroacetic acid (TCA) and thiobarbituric acid (TBA). Then, the macerate was incubated in a thermoblock at 95 °C for 30 min and subsequently centrifuged at 13.000× *g* for 10 min at 4 °C. Absorbance was measured using a Thermo Scientific Spectronic Genesis 10 UV-Vis Scanning Spectrophotometer (Madison, WI, USA) at 532, 600, and 440 nm to correct for interference generated by TBARS-sugar complexes. The results were expressed as nmol of malondialdehyde per gram of fresh weight (MDA nmol g^−1^ FW).

### 2.9. Proline

Proline was determined using the Bates et al. [40] method, which involved grinding 0.3 g of plant material and homogenizing it with 1 mL of 3% sulfosalicylic acid. The supernatant was transferred to a test tube and reacted with 2 mL of acid ninhydrin (1.25% ninhydrin + 80% glacial acetic acid). It was then incubated in an oven at 100 °C for 60 min. After rapid immersion in an ice bath for 10 min to stop the reaction, the absorbance was measured at 520 nm using a Thermo Scientific Spectronic Genesis 10 UV-Vis Scanning Spectrophotometer (Madison, WI, USA).

### 2.10. Experimental Design and Statistical Analysis

The experimental design was completely randomized, with two factors: irrigation (2 levels) and ChNPs dosage (3 levels), and six replicates per treatment (36 experimental units). Three of those units per treatment were used to measure biomass, and three more were used to determine photosynthetic and biochemical parameters. Statistical analysis of the data was performed using Jamovi 2.7.24, with a two-way ANOVA at the 5% significance level (p < 0.05) and, subsequently, a mean-comparison test (Tukey). In addition, Pearson’s and pairwise correlations were performed.

## 3. Results

### 3.1. Characterization of Nanoparticles

**Figure 1:**
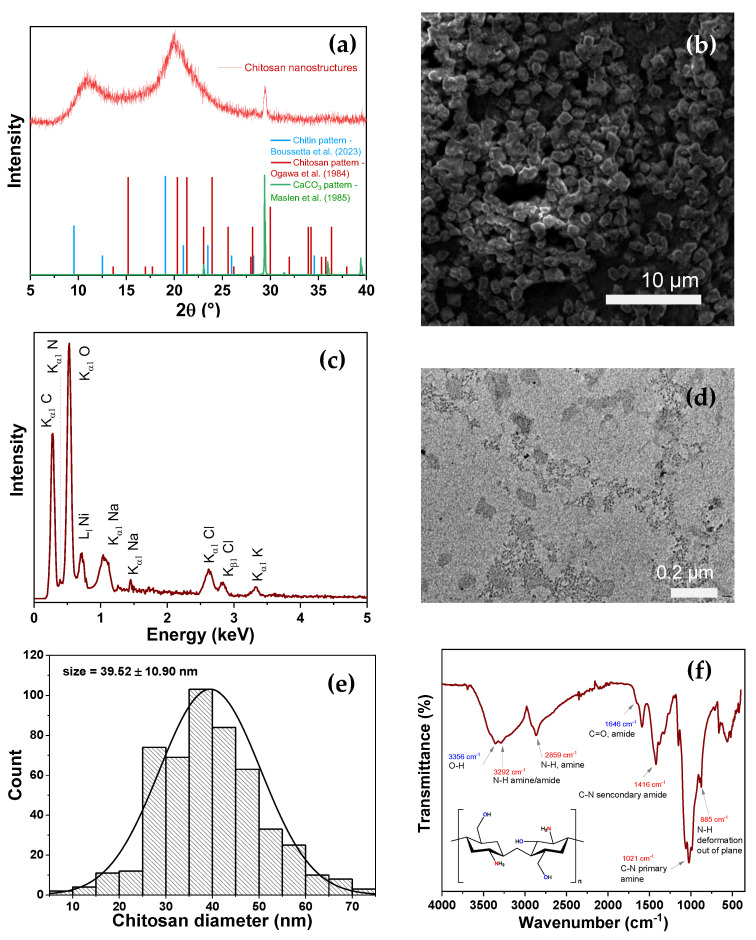
Structural and morphological characterization of chitosan nanostructures. (**a**) X-ray diffraction pattern and standard patterns [43,44,45], (**b**) SEM image, (**c**) SEM-EDX spectrum, (**d**) TEM image, (**e**) size distribution histogram (n = 500), and (**f**) FTIR spectrum.

The use of chitosan in agriculture, whether as a biostimulant or a water-evaporation controller, is limited by its natural sources and degree of deacetylation, which affect its physicochemical properties [41,42]. One of the challenges in scaling up chitosan use is reproducing results across different climates and chitin sources. Characterization of the nanostructures in the samples and their interfaces enables correlation of their effects on photosynthetic biochemistry and performance indicators with their physicochemical properties. Chitosan was characterized by X-ray diffraction, as shown in Figure 1a. The fundamental structure of α-chitin was verified through diffraction angles (2θ) at values of 9.7°, 12.5°, and 19.1°, corresponding to the (020), (021), and (110) planes of the α-chitin primary structure that had undergone deacetylation. The 2θ value of 29.32° is associated with the (104) plane of calcite, CaCO_3_, as a residue of natural biopolymer processing [43]. The calculated size of chitosan crystallites, based on XRD data, was 4.87 nm. On the other hand, the macroscopic chitosan powder was evaluated using SEM images, as shown in Figure 1b. Polyhedral structures that approximate cubic morphology exhibited a characteristic length of 1.27 ± 0.25 μm (n = 500). However, surface irregularities associated with smaller chitosan structures were also observed. The EDX spectrum (Figure 1c) for the selected area revealed the spectroscopic signals of the carbon K_α1_ C: 0.270 eV, nitrogen K_α1_ N: 0.397 keV, and oxygen K_α1_ C: 0.535 keV of forming elements of chitosan. Additionally, the signals of impurities, such as sodium K_α1_ (Na: 1.046 keV) and chlorine K_α1_ K_α1_ (Cl: 2.628 keV), were verified as residuals from the demineralization and deacetylation steps. The signals of aluminum K_α1_ Al: 1.439 keV and nickel LI Ni: 0.723 keV are associated with the conductive tape used for supporting the nanomaterial. The ChNPs were then evaluated by TEM imaging (Figure 1d), which showed their spherical morphology and a mean size of 39.52 ± 10.90 nm (n = 500, histogram, Figure 1e). Infrared spectroscopy (Figure 1f) revealed the characteristic stretching vibrations of chemical groups present in chitosan, enabling a combined analysis with other instrumental techniques. The broad peaks observed in the 3400–3200 cm^−1^ region are attributed to the overlapping stretching vibrations of O-H (hydroxyl, 3355 cm^−1^) and N-H (amine, 3281 cm^−1^) groups and intramolecular hydrogen bonding. The peak at 3281 cm^−1^ coincides with the overlapping N-H stretching vibrations of both amine and amide groups. The peak at 2864 cm^−1^ is associated with the N-H groups of primary amines, as expected following chitin deacetylation. The stretching vibration at 1593 cm^−1^ corresponds to the N-H bending vibrations of the amine group, while a subtle peak at 1646 cm^−1^ indicates the C=O stretching of residual N-acetyl groups from chitin.

### 3.2. Biomass Production

**Figure 2:**
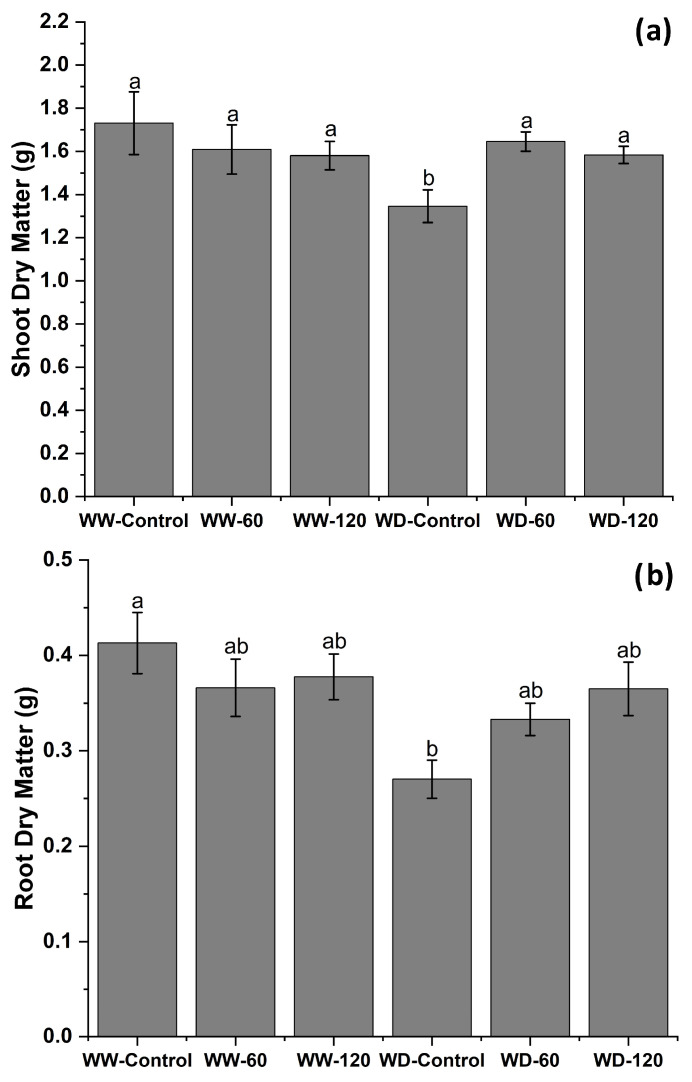
Dry matter production in 40-day-old *S. lycopersicum* seedlings subjected to water deficit and ChNPs (0, 60, and 120 mg L^−1^). (**a**) Shoot dry matter, and (**b**) Root dry matter. Different letters indicate significant differences according to the Tukey test (*p* ≤ 0.05). Means ± SE, n = 3.

A two-way ANOVA revealed a significant interaction (*p* ≤ 0.05) between water availability and ChNPs dosage. The biomass production from shoots and roots was characterized by a significant reduction (*p* ≤ 0.05) in the WD-Control treatment (around 22% and 34% less in shoots and roots, respectively) and subsequent recovery to the level of WW plants in the WD-60 and WD-120 (*p* ≤ 0.05) treatments (Figure 2).

### 3.3. Photosynthetic Performance in S. lycopersicum

**Figure 3:**
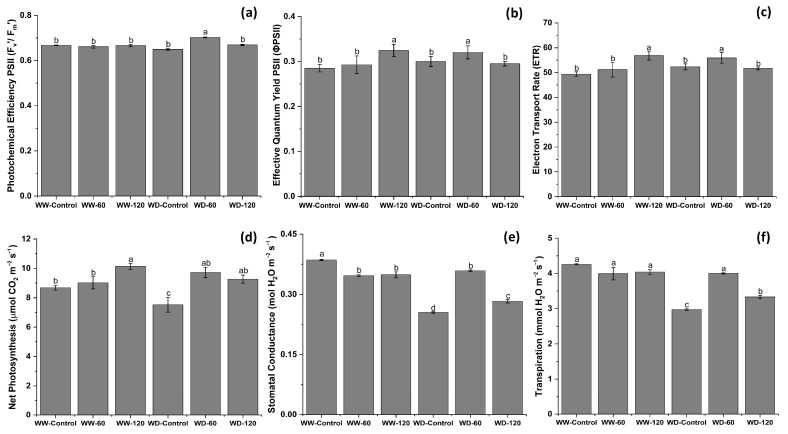
Photosynthetic parameters of 40-day-old *S. lycopersicum* seedlings subjected to water deficit and ChNPs (0, 60, and 120 mg L^−1^). (**a**) Photochemical efficiency of PSII, (**b**) Effective quantum yield of PSII, (**c**) Electron transport rate, (**d**) Net photosynthesis, (**e**) Stomatal conductance, and (**f**) Transpiration. Different letters indicate significant differences according to the Tukey test (*p* ≤ 0.05). Means ± SE, n = 3.

**Figure 4:**
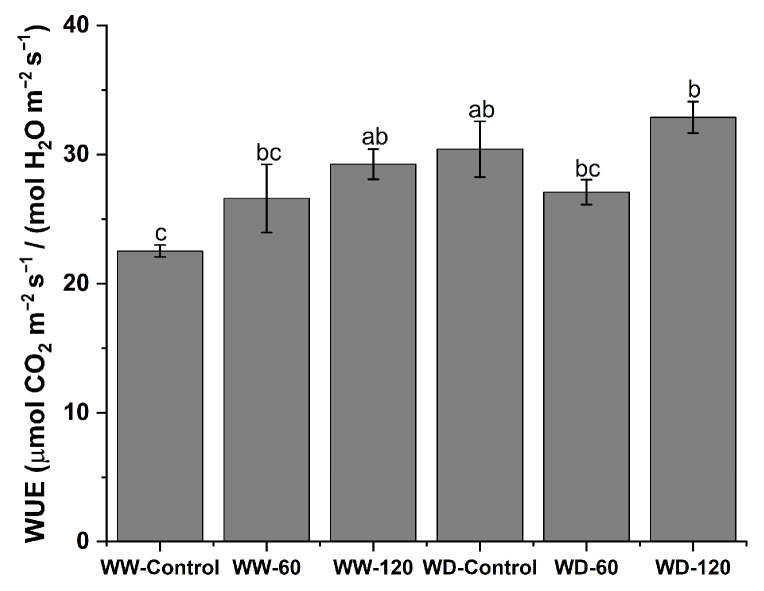
Intrinsic water-use efficiency of 40-day-old *S. lycopersicum* seedlings subjected to water deficit and ChNPs (0, 60, and 120 mg L^−1^). Different letters indicate significant differences according to the Tukey test (*p* ≤ 0.05). Means ± SE, n = 3.

The photosynthetic performance was evaluated with respect to the photochemical phase and gas exchange. All variables showed a significant interaction (*p* ≤ 0.05) between water availability and ChNPs dosage. The photochemical efficiency of photosystem II (F_v_′/F_m_′) was significantly higher in the WD-60 treatment (*p* ≤ 0.05) compared to the WD-Control and other treatments. Similarly, the effective quantum yield of photosystem II (Φ_PSII_) was approximately 14% higher (*p* ≤ 0.05) for WW-120 and WD-60 than for the well-watered control, and approximately 10% higher (*p* ≤ 0.05) than for the water-deficit control. The same tendency was observed in the electron transport rate (ETR), which increased (*p* ≤ 0.05) by 16% and 14% for WW-120 and WD-60, respectively, compared to the WW-Control. The ETR and Φ_PSII_ values for WD-60 were significantly (*p* ≤ 0.05) higher than WD-Control values (Figure 3a–c). Although net photosynthesis (P_n_) was significantly (*p* ≤ 0.05) reduced by water deficit by 15%, it recovered in plants treated with ChNPs, exceeding that of well-watered plants by 11.7% (*p* < 0.05). Furthermore, the values of well-watered plants increased even further with ChNPs. Stomatal conductance (*g*_s_) was strongly affected by water deficit, with a 34% reduction observed in WD-Control compared to WW-Control (*p* ≤ 0.05). However, in the WD-60 treatment, this value decreased by only 7.8%. Transpiration (*E*) followed the same trend as *g*_s_, with a 29% reduction (*p* ≤ 0.05) in WD-Control compared to WW-Control and a recovery to the level of well-watered plants in the WD-60 treatment (Figure 3d–f). The intrinsic water use efficiency (WUE) was higher in the WD-Control and WD-120 treatments (Figure 4). Interestingly, the WD-60 treatment showed values similar to those of the WW-Control treatment (*p* > 0.05). There were no significant differences in the amount of photosynthetic pigments (Chl *a* and Chl *b*) (Table 1); however, the Chl *a*/*b* ratio was lower in well-watered control plants (*p* ≤ 0.05) and higher in well-watered plants treated with ChNPs (*p* ≤ 0.05). Interestingly, this ratio was also higher under water-deficit conditions without ChNPs and was similar to that at the 60 mg L^−1^ dose under water deficit.

### 3.4. Antioxidant System

**Figure 5:**
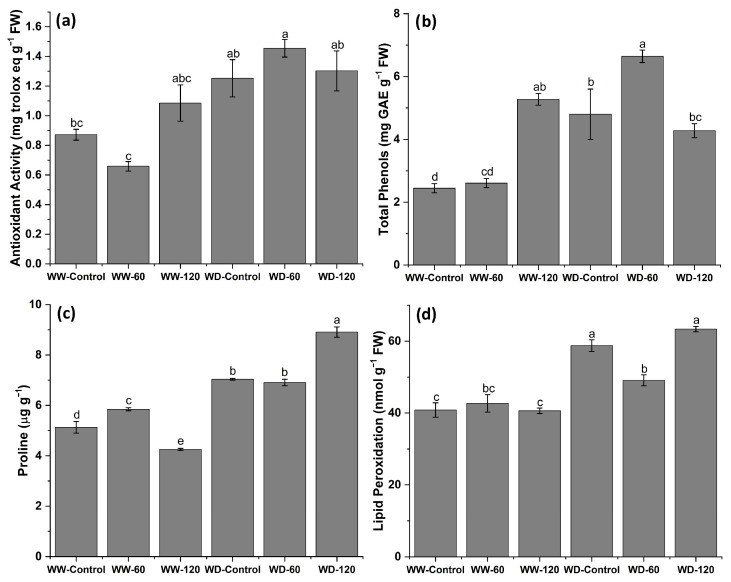
Antioxidant system parameters in leaves of 40-day-old *S. lycopersicum* seedlings subjected to water deficit and ChNPs (0, 60, and 120 mg L^−1^). (**a**) Antioxidant activity, (**b**) Total phenols, (**c**) Proline, and (**d**) Lipid peroxidation. Different letters indicate significant differences according to the Tukey test (*p* ≤ 0.05). Means ± SE, n = 3.

The antioxidant system was evaluated by analyzing antioxidant activity, total phenol and proline levels, and lipid peroxidation. In our study, antioxidant activity was higher in plants subjected to water deficit and treated with ChNPs than in the WW-Control and WW-60 groups (Figure 5a). The highest value was observed in WD-60, which was 66% higher than WW-Control (*p* ≤ 0.05). The response of total phenols was similar to the antioxidant activity (Figure 5b). The lowest values were recorded in WW-Control and WW-60, while the highest was observed in WD-60, which was 1.6-fold greater than WW-Control (*p* ≤ 0.05). Proline levels were lower in WW plants and higher in the WD group (Figure 5c). Interestingly, WD-120 showed the highest value (*p* ≤ 0.05), whereas WD-60 was similar to WD-Control; both were 34% higher than WW-Control. As expected, lipid peroxidation (LP) was 46.7% higher in WD-Control than WW-Control (Figure 5d) (*p* ≤ 0.05). The application of ChNPs in WD-60 reduced LP, whereas WD-120 showed a value comparable to WD-Control.

## 4. Discussion

### 4.1. Chitosan NPs Are Viable for Biotechnological Applications

Nanoparticles have been proposed as an alternative for mitigating abiotic stresses in food production. Among them, chitosan nanoparticles (ChNPs) exhibit biocompatibility, non-toxicity, biodegradability, and low allergenicity, making them useful in a variety of applications, including in agriculture [30]. The ChNPs used in this study do not have the same structure as α-chitin, which exhibits a distinctive diffraction pattern [44]. Zhang et al. [46] reported a progressive shift towards higher 2θ values associated with deacetylation, in which the chitin structure approaches that of chitosan. The characteristic peaks of the chitosan structure were observed at 2θ values of 15.2°, 20.3°, and 21.3°, corresponding to the (120), (022), and (020) planes of the orthorhombic chitosan structure JCPDS # 039-1894, as reported by Ogawa et al. [45] (Figure 1a). The vibrations at 1151 and 1059 cm^−1^ are associated with C-O stretching, particularly in the C-O-C bridge and C-OH groups within the polysaccharide structure [47,48], whilst the vibration at 1023 cm^−1^ is attributed to C-N stretching in primary amines (Figure 1f). These findings collectively confirm significant deacetylation in the material and highlight the characteristics of ChNPs and their viability for biotechnological applications.

### 4.2. Chitosan Nanoparticles Counteract the Harmful Effects of Water Deficit

**Figure 6:**
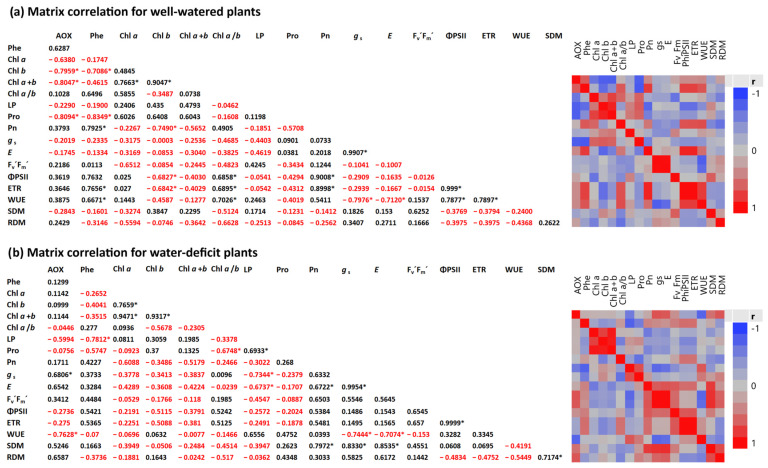
Matrix of Pearson correlation. (**a**) well-watered plants, and (**b**) water-deficient plants. * Indicates statistical significance (*p* < 0.05). Abbreviation: AOX, antioxidant activity; Phe, total phenols; Chl *a*, chlorophyll *a*; Chl *b*, Chlorophyll *b*; Chl *a* + *b*, total chlorophyll; Chl *a*/*b*, chlorophyll *a*/*b* ratio; LP, lipid peroxidation; Pro, proline; P_n_, net photosynthesis; *g*_s_, stomatal conductance; *E*, transpiration; F_v_′/F_m_′, photochemical efficiency of PSII; Φ_PSII_, effective quantum yield of PSII; ETR, electron transport rate; WUE, intrinsic water use efficiency; SDM, shoot dry matter; RDM, root dry matter.

It is well known that water deficit is one of the most significant constraints on crop production worldwide. It has been reported that *S. lycopersicum* is highly sensitive to water deficit, with a yield response factor (Ky) of 1.05 [49]. Water deficit is particularly relevant during the early stages of *S. lycopersicum* cultivation, significantly reducing biomass production [50]. These previous findings are consistent with our results, which showed that treating 50% of the water reduced shoot biomass by around 22% and root biomass by 34% in seedlings (Figure 2). By contrast, the presence of WD and ChNPs, particularly at 60 mg L^−1^, resulted in a full recovery of both shoot and root biomass, reaching levels comparable to those observed in WW plants. Notably, the greater reduction in root biomass (34%) compared to shoot biomass (22%) under WD-Control suggests that roots are disproportionately sensitive to early water restriction—a response consistent with the role of roots as the primary organ sensing soil water availability [49]. The capacity of ChNPs at 60 mg L^−1^ to restore both organs to WW levels implies that their biostimulatory action extends beyond foliar metabolism, potentially influencing root architecture and water-uptake capacity under stress conditions. Our findings are similar to those reported by [27] in the same species, who reported a 38% increase in shoot dry mass in plants treated with ChNPs and exposed to heavy metal stress. Similarly, Dawood et al. [51] and Dashtmian et al. [52] found that foliar applications of ChNPs at 20 mg L^−1^ and 100 mg kg^−1^ increased vegetative and yield parameters in *Vicia faba* L. and *Phaseolus vulgaris* L., further indicating that ChNPs mitigate the adverse effects of water deficit. On the other hand, Attaran et al. [53] reported beneficial effects of 60 and 90 mg kg^−1^ ChNPs on *Salvia abrotanoides* (Kar.) under water deficit, as evidenced by improved photosynthetic performance and antioxidant system activity. Biomass production is mainly driven by primary metabolism and is closely linked to photosynthetic performance. In our case, there was a positive pairwise correlation between P_n_ and shoot dry matter (SDM) under WD, which was significant at 120 mg L^−1^ (r = 0.99, *p* = 0.04; see Table S1). This is consistent with our observations of chlorophyll fluorescence and gas-exchange parameters (F_v_′/F_m_′, Φ_PSII_, ETR, P_n_, *g*_s_, and *E*) in which the presence of WD and ChNPs, particularly at WD-60 mg L^−1^, resulted in parameters similar to those of WW plants, alleviating the consequences of water deficit (Figure 3). Overall, this trend is consistent with the positive Pearson correlations among SDM, RDM, P_n_ (r = 0.79), *g*_s_ (r = 0.83), and *E* (r = 0.85) in plants subjected to WD (Figure 6b). The strength of these correlations reveals that under mild-to-moderate water deficit, biomass accumulation was primarily governed by the maintenance of stomatal conductance and net photosynthesis rather than by antioxidant activity alone. This suggests a hierarchical response: ChNPs first act at the stomatal and photosynthetic level, sustaining carbon assimilation and growth, while the antioxidant system operates as a complementary protective layer, particularly relevant at the 60 mg L^−1^ dose, where lipid peroxidation was simultaneously reduced (Figure 5d). This mechanistic sequence, visible in the correlation structure of Figure 6b, strengthens the interpretation that ChNPs confer drought resilience through an integrated, multi-level physiological response. Our results are consistent with those of Behboudi et al. [32] and Ali et al. [28], who reported positive effects on the photosynthetic rate and biomass of *Triticum aestivum* L. and *Catharanthus roseus* at a concentration of 90 mg kg^−1^ and 10.000 mg L^−1^, respectively.

It is important to note that the water deficit imposed in this study (50% WHC) was intentionally designed as a mild-to-moderate stress, consistent with the irrigation-deficit conditions commonly encountered in early crop management, rather than severe terminal drought. This design choice enabled detection of early physiological responses and biostimulant efficacy before irreversible cellular damage, which is particularly relevant given that *S. lycopersicum* is highly sensitive even to moderate water restrictions [16,49].

On the other hand, an increase in water deficit led to higher antioxidant system parameters and osmoprotectant levels in *S. lycopersicum*, which were triggered as adaptive mechanisms in response to stress [54]. This was clearly observed in our study, particularly in the relationship between antioxidant activity and lipid peroxidation, with a negative pairwise correlation in WD treatments and a positive one in WW plants. This became even more relevant when we noted that these relationships were stronger and more significant with the addition of ChNPs at 120 mg L^−1^ in WD (r = 0.99, *p* = 0.04), revealing an enhanced effect on the plant’s regulatory processes for stress adaptation (Table S1). The same tendency is observed in Figure 6a,b (although not significant) when only the WW and WD conditions are considered. This increase in the non-enzymatic antioxidant component, also observed under ChNP treatment, was reported by Reyes-Pérez et al. [55] in the same species under non-stress conditions.

It has been reported that ChNPs can enhance photosynthesis by increasing the chlorophyll content by 30–50% [53,56]. Surprisingly, no differences were found between the treatments in our study. However, plants treated with ChNPs (excluding WD-120) exhibited a higher Chl *a*/*b* ratio. Based on the inverse linear relationship between photoinhibition and the Chl *a*/*b* ratio reported by Aro et al. [57], this ratio could confer greater photoprotection of photosystem II, and thus explain the improved performance of the photochemical component observed in the treated plants (Figure 3). Additionally, a Pearson correlation was observed between the *a*/*b* ratio and F_v_′/F_m_′ and PSII under WW conditions (Figure 6), although they were not significant in the WD condition. Effects of chitosan on *g*_s_ have been reported across different species, with contrasting results [28]. In all cases, it appears that chitosan is involved in abscisic acid (ABA) signaling, determining stomatal opening or closing. The latter is associated with increased transcription of the *LeNCED1* and *SlAREB1* genes, which catalyze ABA synthesis, as evaluated by Mohamed and Abdel-Hakeem [58] in *S. lycopersicum* under water deficit. These findings are consistent with our observations, as *g*_s_ increased by 41% in WD plants at a dose of 60 mg L^−1^ ChNPs compared with WD plants without ChNPs. In this regard, as Figure 3e shows, *g*_s_ decreases in WW 60 and 120, which can also be explained by the aforementioned effect, as proposed by Priyaadharshini et al. [59]. In this regard, Ali et al. [28] noted that this response may be species-specific, as ChNPs have been shown to increase or decrease ABA concentrations and their effects on stomatal modulation, depending on the species. As a result of these effects, the change in the proportions of *g*_s_ and P_n_ (P_n_/*g*_s_) increased WUE, which was most evident in WW plants treated with ChNPs. Importantly, while WD-Control plants exhibited the highest WUE values—a classical drought response driven by stomatal closure that prioritizes water conservation at the expense of carbon assimilation—WD-60 plants showed WUE values statistically similar to those of WW-Control (*p* > 0.05) (Figure 4). This normalization of WUE under stress is agronomically significant: it indicates that ChNPs at 60 mg L^−1^ restored stomatal aperture sufficiently to sustain photosynthetic productivity without incurring the water-use penalty typically associated with drought-induced stomatal shutdown. This balance between water conservation and carbon gain represents a key trait for crop resilience under mild-to-moderate water deficit. It has been similarly observed by Akhtar et al. [60] in *Calendula officinalis* L. The work of authors Mohamed and Abdel-Hakeem [58] is the only available study to evaluate the effects of ChNPs and water deficit on genes that respond to water deficit, including those involved in water transport, ABA synthesis, and heat shock, in *S. lycopersicum*. However, the authors did not consider physiological or biochemical parameters, which we evaluated and found to be consistent with previous results. These results complement each other and reinforce the idea that ChNPs can confer water-deficit tolerance in this species. Additionally, the positive effects of ChNPs on antioxidant metabolism are well documented: they stimulate antioxidant activity through enzymatic and non-enzymatic mechanisms, reduce oxidative stress, and protect the photosynthetic machinery in plants under abiotic stress [28,51,61].

Proline is a multifunctional amino acid that accumulates under various abiotic stress conditions, including water deficit. Beyond its classical role as an osmolyte, proline functions as a reactive oxygen species (ROS) scavenger, a protein stabilizer, and a key stress-signaling molecule [62]. Under the mild-to-moderate stress conditions of this study, the moderate but statistically significant increase in proline levels is thus more consistent with its antioxidant and signaling roles than with bulk osmotic adjustment, a distinction supported by the negative correlation observed between proline and lipid peroxidation, particularly at 60 mg L^−1^ ChNPs. This interpretation aligns with the understanding that proline’s contribution to osmotic adjustment becomes dominant only under severe dehydration [62]. In contrast, its protective and regulatory functions are activated at early or mild stress stages. It has been reported that the addition of ChNPs increased proline content [51], as observed in our study, further reinforcing its protective role in cells. One way ChNPs may stimulate antioxidant metabolism is by increasing nitric oxide (NO) production in treated plants, as NO plays an important role in plant defense signaling [63]. In addition, Chandra et al. [64] reported increased expression of genes associated with phenolic compounds and antioxidant enzymes in plants treated with ChNPs. This appears to be triggered by the identification of chitin components in membrane complexes by chitin elicitor-binding protein (CEBiP), as described by Kaku et al. [65] in a study using chitin in *Oryza sativa* L. Thus, the molecular basis of the effect of ChNPs on secondary metabolites can be explained.

Responses to ChNPs across different species appear to show an effective limit near 100 mg L^−1^, as evidenced by numerous studies that do not consider higher doses [27,29,32,51,52,53,56,58,60], which may also apply to *S. lycopersicum*. Our study partially corroborates this, as the highest dose (120 mg L^−1^) did not produce the best results for most measured parameters (photochemical and gas exchange, antioxidant activity, and total phenols). In addition, there are signs of toxicity, as evidenced by an increase in proline and lipid peroxidation. The dose-dependent response observed in this study can be interpreted in light of the physicochemical properties of the ChNPs characterized in Section 2.1. The spherical morphology and mean size of 39.52 nm confirmed by TEM (Figure 1d,e) are within the range associated with efficient foliar uptake through stomatal and cuticular pathways [30,31]. Furthermore, the high density of hydroxyl (-OH) and amino (-NH_2_) groups, as confirmed by FTIR (Figure 1f), confers a strong cationic character that facilitates electrostatic interactions with negatively charged plant cell membranes, promoting cellular uptake and intracellular signaling at 60 mg L^−1^. However, at 120 mg L^−1^, this same cationic density may exceed the membrane’s buffering capacity, disrupting lipid bilayer integrity and triggering oxidative stress, as evidenced by increased lipid peroxidation and proline accumulation. This physicochemical explanation links the structural characterization of the nanoparticles to their biological dose-response behavior, reinforcing the importance of rigorous polymer characterization as a prerequisite for safe and effective agricultural applications. A relevant methodological consideration is the absence of fresh weight measurements in this study. While dry matter was chosen as a stable and reproducible indicator of biomass allocation, fresh weight data would have allowed estimation of tissue water content and thus determination of whether significant plant dehydration occurred under the 50% WHC regime. However, given that the imposed water deficit was mild-to-moderate and that stomatal conductance in WD-Control plants, despite being reduced by 34%, remained at levels associated with partial stomatal closure rather than stomatal shutdown, severe cellular dehydration is unlikely to have confounded the fresh-weight-based biochemical variables. Nevertheless, future studies should incorporate water-content measurements and express biochemical variables on a dry-weight basis to enable full comparison across stress intensities. Similarly, measuring additional compatible solutes, such as soluble sugars, would strengthen the characterization of osmotic adjustment mechanisms. These results demonstrate the multifaceted benefits of chitosan application, including enhanced biomass production, improved photosynthetic performance, and an enhanced antioxidant system under limited water availability. The intrinsic structural properties of chitosan, characterized by a high density of hydroxyl (-OH) and amino (-NH_2_) groups, enable it to serve as a dual-action agent: a potent scavenger of reactive oxygen species (ROS) and a sequestrant of metal ions [66]. This chemical buffering likely mitigates oxidative damage at the cellular level. However, to fully elucidate the biostimulatory potential of chitosan, future studies must prioritize the enzymatic components of the antioxidant system and molecular characterization of stress-responsive gene expression pathways. In addition, the present results suggest that nano chitosan can help mitigate water deficits in the Latin American context and could be evaluated in other regions of the world and across species’ life cycles.

## 5. Conclusions

This study demonstrated that mild-to-moderate water deficit (50% WHC) significantly reduced shoot and root biomass by 22% and 34%, respectively, and impaired photosynthetic performance, as evidenced by a 15% reduction in net photosynthesis and a 34% decrease in stomatal conductance, while increasing lipid peroxidation by 46.7% in *S. lycopersicum* seedlings. Foliar application of ChNPs at 60 mg L^−1^ effectively mitigated these adverse effects, restoring biomass production to well-watered levels, improving photochemical efficiency (ΦPSII and ETR by ~14%), increasing net CO_2_ assimilation by 11.7%, and enhancing antioxidant activity and total phenols by 66% and 1.6-fold, respectively. These results position ChNPs as a promising, sustainable biostimulant for early crop management under water-scarce conditions, particularly relevant for tomato-producing regions of Latin America.

Future research should evaluate ChNPs efficacy under more severe or prolonged drought conditions and across the full crop cycle, incorporating enzymatic antioxidant components, quantification of compatible solutes, such as soluble sugars, fresh-weight-based biochemical analyses, and molecular characterization of stress-responsive gene expression pathways. Field-scale validation across different genotypes, climates, and chitin sources will be essential to confirm the translational potential of this approach.

## Figures and Tables

**Table 1:** Photosynthetic pigments in leaves of 40-day-old *S. lycopersicum* seedlings subjected to water deficit and ChNPs (0, 60, and 120 mg L^−1^) expressed in μg g^−1^ FW.

Treatment	Chl *a*	Chl *b*	Chl *a* + *b*	Chl *a*/*b*
WW-Control	10.3 ± 0.23 ^a^	8.1 ± 0.23 ^a^	18.7 ± 0.48 ^a^	13.0 ± 0.016 ^c^
WW-60	11.5 ± 0.09 ^a^	8.2 ± 0.03 ^a^	19.6 ± 0.11 ^a^	13.9 ± 0.005 ^ab^
WW-120	10.5 ± 0.47 ^a^	7.4 ± 0.21 ^a^	17.9 ± 0.68 ^a^	14.1 ± 0.023 ^a^
WD-Control	11.1 ± 0.26 ^a^	8.0 ± 0.33 ^a^	19.1 ± 0.59 ^a^	13.8 ± 0.024 ^ab^
WD-60	10.5 ± 0.23 ^a^	7.6 ± 0.12 ^a^	18.2 ± 0.35 ^a^	13.6 ± 0.010 ^abc^
WD-120	10.6 ± 0.05 ^a^	8.0 ± 0.04 ^a^	18.7 ± 0.03 ^a^	13.1 ± 0.011 ^bc^

Different letters in the same column indicate significant differences according to the Tukey test (*p* ≤ 0.05).

## Data Availability

The raw data supporting the conclusions of this article will be made available by the authors on request.

## Supplementary Materials

The following supporting information can be downloaded at: https://www.mdpi.com/article/10.3390/polym18111275/s1, Table S1: Supplementary Table S1. Pairwise correlation.

## Abbreviations

The following abbreviations are used in this manuscript:

Phe	Total phenols
Chl *a*	Chlorophyll *a*
Chl *b*	Chlorophyll *b*
Chl *a* + *b*	Total chlorophyll
Chl *a*/*b*	Chlorophyll a/b ratio
LP	Lipid peroxidation
Pro	Proline
P_n_	Net photosynthesis
*g*_s_	Stomatal conductance
*E*	Transpiration
F_v_′/F_m_′	Photochemical efficiency of PSII
Φ_PSII_	Effective quantum yield of PSII
ETR	Electron transport rate
WUE	Intrinsic water use efficiency
SDM	Shoot dry matter
RDM	Root dry matter

## References

[1] Farooq M., Hussain M., Wahid A., Siddique K.H.M., Aroca R. (2012). Drought stress in plants: An overview. Plant Responses to Drought Stress.

[2] Satoh Y., Yokohata T., Pokhre Y., Hanasaki N., Boulange J., Burek P., Veldkam T., Takata K., Shiogama H. Multi-type global drought projection using multi-model hydrological simulations. Proceedings of the EGU2020 General Assembly Conference Abstracts.

[3] Peña-Guerrero M.D., Nauditt A., Muñoz-Robles C., Ribbe L., Meza F. (2000). Drought impacts on water quality and potential implications for agricultural production in the Maipo River Basin, Central Chile. Hydrol. Sci. J..

[4] Aitken D., Rivera D., Godoy-Faúndez A., Holzapfel E. (2016). Water scarcity and the impact of the mining and agricultural sectors in Chile. Sustainability.

[5] Freedman H., AghaKouchak A., Rigden A.J., Hoek A.V.D., Tomlinson B. (2025). Disparities in the impact of drought on agriculture across countries. Sci. Rep..

[6] Jangid K., Dwivedi P. (2016). Physiological responses of drought stress in tomato: A review. Int. J. Agric. Environ. Biotechnol..

[7] Ahanger M., Qi M., Huang Z., Xu X., Begum N., Qin C., Zhang C., Ahmad N., Mustafa N., Ashraf M. (2021). Improving growth and photosynthetic performance of drought stressed tomato by application of nano-organic fertilizer involves up-regulation of nitrogen, antioxidant and osmolyte metabolism. Ecotoxicol. Environ. Saf..

[8] Ali M., Sina A., Khandker S., Neesa L., Tanvir E., Kabir A., Khalil M., Gan S. (2021). Nutritional composition and bioactive compounds in tomatoes and their impact on human health and disease: A review. Foods.

[9] FAO (2024). Statistical Data of Food and Agriculture Organization. https://www.fao.org/faostat/es/#data/QCL.

[10] (2024). Observatory of Economic Complexity (OEC) Database. Tomatoes. https://oec.world/en/profile/hs/tomatoes.

[11] Conti V., Romi M., Parri S., Aloisi I., Marino G., Cai G., Cantini C. (2021). Morpho-Physiological classification of Italian tomato cultivars (*Solanum lycopersicum* L.) according to drought tolerance during vegetative and reproductive growth. Plants.

[12] Altaf M., Shahid R., Ren M., Naz S., Altaf M., Khan L., Tiwari R., Lal M., Shahid M., Kumar R. (2022). Melatonin improves drought stress tolerance of tomato by modulating plant growth, root architecture, photosynthesis, and antioxidant defense system. Antioxidants.

[13] Arbona V., Ximénez-Embún M.G., Echavarri-Muñoz A., Martin-Sánchez M., Gómez-Cadenas A., Ortego F., González-Guzmán M. (2020). Early molecular responses of tomato to combined moderate water stress and tomato red spider mite *Tetranychus evansi* attack. Plants.

[14] Thole V., Vain P., Martin C. (2021). Effect of elevated temperature on tomato post-harvest properties. Plants.

[15] Delgado-Vargas V.A., Ayala-Garay O.J., Arévalo-Galarza M.D.L., Gautier H. (2023). Increased temperature affects tomato fruit physicochemical traits at harvest depending on fruit developmental stage and genotype. Horticulturae.

[16] Cui J., Shao G., Lu J., Keabetswe L., Hoogenboom G. (2019). Yield, quality and drought sensitivity of tomato to water deficit during different growth stages. Sci. Agric..

[17] Flexas J., Escalona J., Medrano H. (1999). Water stress induces different levels of photosynthesis and electron transport rate regulation in grapevines. Plant Cell Environ..

[18] Chaitanya K., Jutur P., Sundar D., Ramachandra A. (2003). Water stress effects on photosynthesis in different mulberry cultivars. Plant Growth Regul..

[19] Gao Y., Xia J., Chen Y., Zhao Y., Kong Q., Lang Y. (2016). Effects of extreme soil water stress on photosynthetic efficiency and water consumption characteristics of *Tamarix chinensis* in China’s Yellow River Delta. J. For. Res..

[20] Galmés J., Conesa M., Ochogavía J., Perdomo J., Francis D., Ribas-Carbó M., Savé R., Flexas J., Medrano H., Cifre J. (2011). Physiological and morphological adaptations in relation to water use efficiency in Mediterranean accessions of *Solanum lycopersicum*. Plant Cell Environ..

[21] Liang G., Liu J., Zhang J., Guo J. (2020). Effects of drought stress on photosynthetic and physiological parameters of tomato. J. Am. Soc. Hortic. Sci..

[22] Sperdouli I., Mellidou I., Moustakas M. (2021). Harnessing chlorophyll fluorescence for phenotyping analysis of wild and cultivated tomato for high photochemical efficiency under water deficit for climate change resilience. Climate.

[23] Conti V., Mareri L., Faleri C., Nepi M., Romi M., Cai G., Cantini C. (2019). Drought stress affects the response of Italian local tomato (*Solanum lycopersicum* L.) varieties in a genotype-dependent manner. Plants.

[24] Reddy N., Yang Y., Reddy N., Yang Y. (2015). Introduction to chitin, chitosan, and alginate fibers. Innovative Biofibers from Renewable Resources.

[25] Miliordos D.E., Alatzas A., Kontoudakis N., Kouki A., Unlubayir M., Gémin M.P., Tako A., Hatzopoulos P., Lanoue A., Kotseridis Y. (2022). Abscisic acid and chitosan modulate polyphenol metabolism and berry qualities in the domestic white-colored cultivar savvatiano. Plants.

[26] Sun W., Shahrajabian M.H., Petropoulos S.A., Shahrajabian N. (2023). Developing sustainable agriculture systems in medicinal and aromatic plant production by using chitosan and chitin-based biostimulants. Plants.

[27] Faizan M., Rajput V., Al-Khuraif A., Arshad M., Minkina T., Sushkova S., Yu F. (2021). Effect of foliar fertigation of chitosan na-noparticles on cadmium accumulation and toxicity in *Solanum lycopersicum*. Biology.

[28] Ali E., El-Shehawi A., Ibrahim O., Abdul-Hafeez E., Moussa M., Hassan F. (2021). A vital role of chitosan nanoparticles in improvisation the drought stress tolerance in *Catharanthus roseus* (L.) through biochemical and gene expression modulation. Plant Physiol. Biochem..

[29] Bakhoum G., Sadak M., Tawfic M. (2022). Chitosan and chitosan nanoparticle effect on growth, productivity and some biochemical aspects of *Lupinus termis* under drought conditions. Egypt. J. Chem..

[30] Ingle P.U., Shende S.S., Shingote P.R., Mishra S.S., Sarda V., Wasule D.L., Rajput V.D., Minkina T., Rai M., Sushkova S. (2022). Chitosan nanoparticles (ChNPs): A versatile growth promoter in modern agricultural production. Heliyon.

[31] Wang X., He M., Wang X., Liu S., Luo L., Zeng Q., Wu Y., Zeng Y., Yang Z., Sheng G. (2024). Emerging nanochitosan for sustainable agriculture. Int. J. Mol. Sci..

[32] Behboudi F., Tahmasebi-Sarvestani Z., Kassaee M.Z., Modarres-Sanavy S.A.M., Sorooshzadeh A., Mokhtassi-Bidgoli A. (2019). Evaluation of chitosan nanoparticles effects with two application methods on wheat under drought stress. J. Plant Nutr..

[33] Machado J., Vasconcelos M.W., Soares C., Fidalgo F., Heuvelink E., Carvalho S.M. (2023). Young tomato plants respond differently under single or combined mild nitrogen and water deficit: An insight into morphophysiological responses and primary metab-olism. Plants.

[34] Maxwell K., Johnson G.N. (2000). Chlorophyll fluorescence a practical guide. J. Exp. Bot..

[35] Farquhar G.D., Hubick K.T., Condon A.G., Richards R.A., Rundel P.W., Ehleringer J.R., Nagy K.A. (1989). Carbon isotope fractionation and plant water-use efficiency. Stable Isotopes in Ecological Research.

[36] Lichtenthaler H.K., Wellburn A.R. (1983). Determinations of total carotenoids and chlorophylls *a* and *b* of leaf extracts in different solvents. Biochem. Soc. Trans..

[37] Chinnici F., Bendini A., Gaiani A., Riponi C. (2024). Radical scavenging activities of peels and pulps from cv. Golden Delicious apples as related to their phenolic composition. J. Agric. Food Chem..

[38] Singleton V.L., Rossi J.A. (1965). Colorimetry of total phenolics with phosphomolybdic-phosphotungstic acid reagents. Am. J. Enol. Vitic..

[39] Du Z., Bramlage W.J. (1992). Modified thiobarbituric acid assay for measuring lipid oxidation in sugar-rich plant tissue extracts. J. Agric. Food Chem..

[40] Bates L., Waldren R., Teare I. (1973). Rapid determination of free proline for water-stress studies. Plant Soil.

[41] Román-Doval R., Torres-Arellanes S.P., Tenorio-Barajas A.Y., Gómez-Sánchez A., Valencia-Lazcano A.A. (2023). Chitosan: Properties and its application in agriculture in context of molecular weight. Polymers.

[42] Montes-Ramírez P., Montaño-Leyva B., Blancas-Benitez F.J., Bautista-Rosales P.U., Ruelas-Hernández N.D., Martínez-Robinson K., González-Estrada R.R. (2024). Active films and coatings based on commercial chitosan with natural extracts addition from coconut by-products: Physicochemical characterization and antifungal protection on tomato fruits. Food Control.

[43] Maslen E.N., Streltsov V.A., Streltsova N.R., Ishizawa N. (1995). Electron density and optical anisotropy in rhombohedral carbonates. III. Synchrotron X-ray studies of CaCO_3_, MgCO_3_ and MnCO_3_. Struct. Sci..

[44] Boussetta A., Benhamou A.A., Charii H., Ablouh E.H., Mennani M., Kasbaji M., Boussetta N., Grimi N., Moubarik A. (2023). Formulation and characterization of chitin-starch bio-based wood adhesive for the manufacturing of formaldehyde-free composite particleboards. Waste Biomass Valoris..

[45] Ogawa K., Hirano S., Miyanishi T., Yui T., Watanabe T. (1984). A new polymorph of chitosan. Macromolecules.

[46] Zhang Y., Xue C., Xue Y., Gao R., Zhang X. (2005). Determination of the degree of deacetylation of chitin and chitosan by X-ray powder diffraction. Carbohydr. Res..

[47] Gondim B.L.C., Castellano L.R.C., de Castro R.D., Machado G., Carlo H.L., Valença A.M.G., de Carvalho F.G. (2018). Effect of chitosan nanoparticles on the inhibition of *Candida* spp. Biofilm on denture base surface. Arch. Oral Biol..

[48] Menezes J.E.S.A., Dos Santos H.S., Ferreira M.K.A., Magalhães F.E.A., Da Silva D.S., Bandeira P.N., Saraiva G.D., Pessoa O.D.L., Ricardo N.M.P.S., Cruz B.G. (2020). Preparation, structural and spectroscopic characterization of chitosan membranes containing allantoin. J. Mol. Struct..

[49] Kamanga R., Mbega E., Ndakidemi P. (2018). Drought tolerance mechanisms in plants: Physiological responses associated with water deficit stress in Solanum lycopersicum. Adv. Crop Sci. Technol..

[50] Patane C., Tringali S., Sortino O. (2011). Effects of deficit irrigation on biomass, yield, water productivity and fruit quality of processing tomato under semi-arid mediterranean climate conditions. Sci. Hortic..

[51] Dawood M.G., El-Awadi M.E.S., Sadak M.S. (2024). Chitosan and its nanoform regulates physiological processes and antioxidant mechanisms to improve drought stress tolerance of *Vicia faba* plant. J. Soil Sci. Plant Nutr..

[52] Dashtmian A., Mazinani S.M., Pazoki A. (2023). Exogenous chitosan nanoparticles modulated drought stress through changing yield, biochemical attributes, and fatty acid profile of common bean (*Phaseolus vulgaris* L.) cultivars. Gesunde Pflanz..

[53] Attaran D.S., Karimian Z., Mostafaei D.M., Samiei L. (2022). Chitosan nanoparticles improve physiological and biochemical responses of *Salvia abrotanoides* (Kar.) under drought stress. BMC Plant Biol..

[54] Niyazova N.N., Huseynova I.M. (2024). The antioxidant defense system of tomato (*Solanum lycopersicum* L.) varieties under drought stress and upon post-drought rewatering. Biochemistry.

[55] Reyes-Pérez J.J., Llerena-Ramos L.T., Tezara W., Reynel V., Hernández-Montiel L.G., Juárez-Maldonado A. (2025). Chitosan application improves the growth and physiological parameters of tomato crops. Horticulturae.

[56] Van S.N., Minh H.D., Anh D.N. (2013). Study on chitosan nanoparticles on biophysical characteristics and growth of *Robusta coffee* in greenhouse. Biocatal. Agric. Biotechnol..

[57] Aro E.M., McCaffery S., Anderson J.M. (1993). Photoinhibition and D1 protein degradation in peas acclimated to different growth irradiances. Plant Physiol..

[58] Mohamed N.G., Abdel-Hakeem M.A. (2023). Chitosan nanoparticles enhance drought tolerance in tomatoes (*Solanum lycopersicum* L.) via gene expression modulation. Plant Gene.

[59] Priyaadharshini M., Sritharan N., Senthil A., Marimuthu S. (2019). Physiological studies on effect of chitosan nanoemulsion in pearl millet under drought condition. J. Pharmacogn. Phytochem..

[60] Akhtar G., Faried H.N., Razzaq K., Ullah S., Wattoo F.M., Shehzad M.A., Sajjad Y., Ahsan M., Javed T., Dessoky E. (2022). Chitosan-induced physiological and biochemical regulations confer drought tolerance in pot marigold (*Calendula officinalis* L.). Agronomy.

[61] Arif Y., Siddiqui H., Hayat S., Faizan M., Hayat S., Yu F. (2022). Role of chitosan nanoparticles in regulation of plant physiology under abiotic stress. Sustainable Agriculture Reviews 53: Nanoparticles: A New Tool to Enhance Stress Tolerance.

[62] Szabados L., Savouré A. (2010). Proline: A multifunctional amino acid. Trends Plant Sci..

[63] Manjunatha G., Niranjan-Raj S., Prashanth G.N., Deepak S., Amruthesh K.N., Shetty H.S. (2009). Nitric oxide is involved in chitosan-induced systemic resistance in pearl millet against downy mildew disease. Pest Manag. Sci..

[64] Chandra S., Chakraborty N., Dasgupta A., Sarkar J., Panda K., Acharya K. (2015). Chitosan nanoparticles: A positive modulator of innate immune responses in plants. Sci. Rep..

[65] Kaku H., Nishizawa Y., Ishii-Minami N., Akimoto-Tomiyama C., Dohmae N., Takio K., Minami E., Shibuya N. (2006). Plant cells recognize chitin fragments for defense signaling through a plasma membrane receptor. Proc. Natl. Acad. Sci. USA.

[66] Dou H.Y., Chen X.Q., Li Z.Q. (2013). A review on the use of chitosan and its derivatives as radical scavenger and metal ions chelating agent. Adv. Mater. Res..

